# Sleep and work functioning in nurses undertaking inpatient shifts in a blue-depleted light environment

**DOI:** 10.1186/s12912-022-00973-4

**Published:** 2022-07-18

**Authors:** Kaia Kjørstad, Patrick M. Faaland, Børge Sivertsen, Håvard Kallestad, Knut Langsrud, Daniel Vethe, Cecilie L. Vestergaard, Anette Harris, Ståle Pallesen, Jan Scott, Øystein Vedaa

**Affiliations:** 1 Department of Mental Health, Norwegian University of Science and Technology, Trondheim, Norway; 2 Department of Research and Development, St. Olavs University Hospital, Trondheim, Norway; 3 Department of Health Promotion, Norwegian Institute of Public Health, Zander Kaaes gt. 7, 5015 Bergen, Norway; 4 Department of Research and Innovation, Helse-Fonna, Haugesund, HF Norway; 5 Department of Psychosocial Science, University of Bergen, Bergen, Norway; 6 Norwegian Competence Center for Sleep Disorders, Haukeland University Hospital, Bergen, Norway; 7 Optentia, The Vaal Triangle Campus of the North-West University, Vanderbijlpark, South Africa; 8 Institute of Neuroscience, Newcastle University, Newcastle upon Tyne, UK

**Keywords:** Blue-depleted light environment, Hospital lighting, Shift work, Sleep, Work function

## Abstract

**Background:**

Blue-depleted light environments (BDLEs) may result in beneficial health outcomes for hospital inpatients in some cases. However, less is known about the effects on hospital staff working shifts. This study aimed to explore the effects of a BDLE compared with a standard hospital light environment (STLE) in a naturalistic setting on nurses’ functioning during shifts and sleep patterns between shifts.

**Methods:**

Twenty-five nurses recruited from St. Olavs Hospital in Trondheim, Norway, completed 14 days of actigraphy recordings and self-reported assessments of sleep (e.g., total sleep time/sleep efficiency) and functioning while working shifts (e.g., mood, stress levels/caffeine use) in two different light environments. Additionally, participants were asked to complete several scales and questionnaires to assess the symptoms of medical conditions and mental health conditions and the side effects associated with each light environment.

**Results:**

A multilevel fixed-effects regression model showed a within-subject increase in subjective sleepiness (by 17%) during evening shifts in the BDLE compared with the STLE (*p* = .034; Cohen’s *d* = 0.49) and an 0.2 increase in number of caffeinated beverages during nightshifts in the STLE compared with the BDLE (*p* = .027; Cohen’s *d* = 0.37). There were no significant differences on any sleep measures (either based on sleep diary data or actigraphy recordings) nor on self-reported levels of stress or mood across the two conditions. Exploratory between-group analyses of questionnaire data showed that there were no significant differences except that nurses working in the BDLE reported perceiving the lighting as warmer (*p* = .009) and more relaxing (*p* = .023) than nurses working in the STLE.

**Conclusions:**

Overall, there was little evidence that the change in the light environment had any negative impact on nurses’ sleep and function, despite some indication of increased evening sleepiness in the BDLE. We recommend further investigations on this topic before BDLEs are implemented as standard solutions in healthcare institutions and propose specific suggestions for designing future large-scale trials and cohort studies.

**Trial registration:**

The study was registered before data collection was completed on the ISRCTN website (ISRCTN21603406).

**Supplementary Information:**

The online version contains supplementary material available at 10.1186/s12912-022-00973-4.

## Background

Exposure to light and darkness over the course of a day is the major cue for entrainment of sleep and wakefulness in humans [1]. However, in modern societies, humans are frequently exposed to artificial light sources during the dark period (evenings and nights) of the day. Artificial or polychromatic white light has been found to aid vision and enhance alertness and performance at night [2, 3]. It is also well established that exposure to artificial light can compromise the rhythmicity and timing of individual sleep and wakefulness patterns and that irregular timing of sleep and wakefulness is associated with sleep problems, medical problems, and mental health problems [4]. More specifically, light exposure can compromise the regularity of the biologically dependent component of an individual’s endogenous circadian rhythm (as opposed to an individual’s behaviorally dependent timing of sleep and wakefulness) and can thus lead to more irregular and/or fragmented sleep, which in turn increases the risk for impaired health or daytime functioning [5]. This is especially relevant for shift workers, who are regularly required to be awake and active during the dark period of the day and who subsequently sleep or rest during the light part of the day. Such rest-activity patterns are associated with an increased risk of for example insomnia or shift work disorder [6–8], cardiovascular disease [9, 10], cancer [11–14], gastrointestinal disorders [15], metabolic disturbances [16], diabetes [17–19], and impaired reproductive health [20–22] as well having adverse effects on mental health [4, 23, 24] and the work-life balance [25].

Shift work is particularly common in the healthcare sector, where 24-h services are necessary to provide required health services [26]. Given the critical need to provide medical care around the clock, it is not surprising that hospital inpatients experience disruptions in their sleep–wake cycles and, thus, typically sleep poorly due to elevated levels of light and noise [27, 28]. In recent years, several clinical and research groups have advocated the installation of blue-depleted light environments (BDLEs; indoor lighting blocking short-frequency, blue light < 530 nm) to counteract the effects of artificial light exposure at night and help stabilize the sleep–wake patterns of hospital inpatients with positive effects being reported following such interventions [29, 30]. Likewise, a study on healthy adults residing in a BDLE found positive effects on sleep without any adverse effects or side effects [31].

Recently, a new acute psychiatric unit was built at St. Olavs Hospital, Østmarka, in Trondheim, Norway (latitude ~ 63°N) in which new lighting systems were installed. The unit consists of two inpatient wards: one ward allows for the introduction of a BDLE during evenings and nights, whereas the other has a standard hospital lighting environment (STLE). This setup was primarily established to investigate the effects of evening and night BDLE on sleep and recovery time for patients admitted to the hospital [32]. However, considering that BDLEs could be implemented across multiple healthcare settings in the future, investigating whether exposure to a BDLE represents any benefit or harm to the nurses engaged in shift work under such conditions warrants the inclusion of a wide range of outcomes. To the best of our knowledge, no previous studies have explored whether working in a BDLE compared with a STLE impacts the work performance and/or well-being of nurses in a naturalistic setting. The main aim of the present study was, therefore, to use both work and sleep diaries and actigraphy recordings to investigate nurses’ sleep patterns, work functioning, levels of stress, and mood state over a 2-week period during which they undertook shifts in either a BDLE or a STLE. Both sleep diaries and actigraphy recordings were included in the study as they are complementary measures (subjective and activity based) of sleep and, as the Actiwatches used in the present study did not measure light exposure (which could be used for estimation of time for lights off), the sleep diaries were used to manually augment the quality of the actigraphy data [33]. The secondary aim was to explore the nurses’ self-reported medical and mental health when working in each light environment.

## Methods

### Study design and participants

The present study was a non-randomized cross-sectional study investigating the association between evening and night BDLE compared with STLE on sleep and work functioning in a sample of nursing staff (nurses and nurse assistants—from now on referred to as nurses) working shifts in an acute psychiatric unit at St. Olavs Hospital (ISRCTN21603406). The psychiatric unit was built as two separate wards with mirror-image layouts each consisting of 20 patient rooms and common areas. In one of the wards, both light fixtures and incident light were depleted of blue light frequencies (< 530 nm) from 18:30 to 07:00, whereas the other ward had standard hospital lighting. Light measurements demonstrated that the lighting in the two wards had similar levels of photopic lux but that the levels of melanopic lux were lower in the BDLE than in the STLE. Details of the layout, light system, and light measurement methods are thoroughly described elsewhere [31]. The nurses could not manually change the lighting manually in any of the light environments, and both units had similar light environments during daytime.

Nurses employed at the acute psychiatric unit at St. Olavs Hospital when the study started in November 2018 were invited to participate if they currently worked at least 50% of fulltime equivalent. No other exclusion criteria were applied. Based on this criterion, 25 of 106 employees were excluded from participation because they were on leave (maternity leave or sick leave). As such, the sample comprised a convenience sample of nurses (*n* = 86) studied in their natural work setting. Study participation was voluntary, and participants were not reimbursed for their participation. When data collection began, one-half of the nursing staff initially worked in the unit with BDLE and the other half in the unit with STLE. The order of the conditions was not randomized due to a preset work schedule in which the nurses rotated between wards every 6 weeks. Some nurses were permanently assigned to work night shifts, whereas others rotated between day and evening shifts or worked weekends only.

Of the 86 nurses who met the inclusion criteria, 25 (29.1%) agreed to participate and signed an informed consent form before participating in the study and providing data during the first round of data collection (while working either in the BDLE (*n* = 12) or STLE (*n* = 13) for 6 weeks). In the second round of data collection, 12 (14.0%) participants provided data from the other light environment (either BDLE (*n* = 6) or STLE (*n* = 6)). Actigraphy data was collected from 23 of 25 participants during the first round of data collection and from 8 of 10 participants in the second round of data collection. Individuals without any actigraphy recordings were excluded from the analyses of actigraphy data. During each round of data collection, participants were provided with an envelope containing an Actiwatch, hard copies of the work diary, the sleep diary, and questionnaires about medical and mental health, and side effects associated with the light environment they currently were working in (see assessments described below for further details). They were asked to keep the diaries and wear the Actiwatch for 14 consecutive days towards the end of each round of data collection and complete the questionnaires at home during before returning the envelope to a locked post box placed in each ward.

The study was designed and implemented by external researchers (i.e., not employed at the acute psychiatric unit at St. Olavs Hospital) in response to concerns raised by nurses and safety representatives about the possible negative side effects of working in a BDLE. The topic was generally discussed with employees in the acute psychiatric unit through informational meetings and one-on-one meetings (e.g., between a nurse and a safety representative or a nurse and management) throughout the planning period, and both nurses and safety representatives were closely involved in the development of the present study. Anonymity was strictly implemented to protect the privacy of those who chose to participate. Given these precautions, it is not clear why the participation rate was not higher. Due to the comparatively small sample size, we were limited in the analyses we were able to conduct, and we were not able to carry out the investigation based on a cross-over design.

### Ethics

The study protocol was approved by the Regional Ethical Committee of Central Norway (REK reference number: 2018/1516). The study was registered on 28/12/2018 through the ISRCTN website (ISRCTN21603406).

### Assessments

- Demographic and background variables—Information was collected regarding sex, age, cohabitation status, whether the nurses had children living at home, number of years worked as nursing staff, and percentage of fulltime equivalent.
- Work diary—The work diary comprised 10 questions to gather data on the day-to-day shift schedule of the participants for 14 consecutive days: the date of the shift and work hours. Evening shifts lasted from 14:30–22:00 and night shifts from 21:45–07:45. They were also asked to report on outcomes that are expected to differ from day-to-day and thus reflect variations in behavior and functioning: the number of caffeinated beverages consumed during each shift (coffee, tea, energy drinks, etc.), levels of sleepiness (from not at all sleepy (1) to very sleepy (5)), stress (from not at all stressed (1) to very stressed (5)), and mood, i.e., positive feelings (from not at all positive (1) to very positive (5)) and negative feelings (from not at all negative (1) to very negative (5)). The work diary was filled out at home after the end of each shift.
- Sleep diary – Sleep was assessed by a sleep diary [34] to provide subjective, daily estimates of sleep patterns for 14 consecutive days (in parallel with the work diary). The following measures were derived from the diary: Time in Bed (TIB), Sleep Period Time (SPT; time between falling asleep and final awakening); Total Sleep Time (TST), Sleep Onset Latency (SOL), Wake After Sleep Onset (WASO), Early Morning Awakening (EMA; time spent in bed after final wake-up time), Sleep Efficiency (SE; total sleep time as a percentage of time in bed), number of awakenings during sleep, and an overall rating of the sleep quality (from very restless (1) to very sound (5)). The sleep diary was filled out after each sleep period.
- Actigraphy – Motor activity was assessed using actigraphy data collected with GENEActive® Actiwatches for 14 consecutive days to derive the following estimates: Wake After Sleep Onset (WASO), Total Sleep Time (TST), Sleep Period Time (SPT; time between falling asleep and the final awakening), and number of sleep periods (SIBS). The Actiwatch data were processed and scored using the GGIR package (version 2.2–1) [35–37] for R (version 3.6.2). The GGIR sleep-detection algorithm was used to infer sleep onset and wakeup-times and to score sleep and wakefulness between these timepoints. Due to a high proportion of daytime sleep in the shift-working participants and incidences of multiple sleep periods within 24 h, the actigraphy output was manually compared with participants’ sleep diaries to correct obvious error estimates by the software. Where obvious discrepancies between sleep diaries and the actigraphy scores appeared, the actigraphy data were replaced by sleep diary data before final calculation of actigraphic sleep variables as recommended in the literature [33].
- Questionnaire data—Participants were asked to complete several scales and questionnaires with adequate psychometric properties to assess medical and mental health, and side effects associated with the light environments. Specifically, the Kessler Psychological Distress Scale (K10; used to identify adults with varying levels of psychological distress) [38], the Psychological Health Questionnaire (PHQ; to assess sleep disturbances, headaches, respiratory infections, and gastrointestinal problems) [39], the Headache and Eyestrain Scale (H&ES; to assess eye strain and headache) [40], an evaluation about beliefs about the light condition (BAL; rating pleasantness and color of the lighting) [41], one additional item probing the experienced adequacy of the lighting in a work setting (‘unsuitable for work/suitable for work’), one item assessing work strain and three items assessing performance and effort from the Psychological Variables Questionnaire [42], and 12 items assessing negative side effects of the light conditions [32].

### Statistical analysis

Fixed-effects regression models were fitted to capture the within-subject effects, using methods of maximum likelihood estimation in STATA version 17 [43]. Sleep- and work diary data, and actigraphy data were structured so that each participant was compared with themselves in terms of how they slept and functioned across the two different light conditions. Sleep periods that started within 15 h after a shift ended were included in the analyses. As previously mentioned, the participants kept diaries and wore an Actiwatch while working in each light condition and typically had ~ 5 shifts during this time. By structuring the data this way, each participant contributed multiple observations across the two light conditions (168 shifts in total), giving the study an acceptable statistical power. Results are shown separately for comparisons of evening BDLE with evening STLE, of night BDLE with night STLE, and of combined evening and night BDLE with combined evening and night STLE. Additionally, mean values for each light environment, estimated mean difference, confidence intervals (CI; 95%), and effects sized in terms of Cohen’s *d* are shown. Cohen’s *d* was calculated in line with recognized guidelines [44, 45]. As a benchmark for interpreting Cohen’s *d*, 0.80 is regarded as large, 0.50 as moderate, and 0.20 as small [46]. Day shifts were not included in the analyses.

Due to the limited sample size of the present study, analyses performed on outcome variables from the questionnaires (i.e., outcomes without multiple observations) should be regarded as exploratory. One-way between-group ANOVAs were performed using IBM SPSS Statistics version 25 [45] to examine differences in the medical and mental health of participants working in the BDLE compared with the STLE during the first round of data collection (data from the second round were excluded due to the inadequate sample size).

## Results

### Sample characteristics

The nurses included in the study had a mean age of 39.9 years (*SD* = 12.3 years), were predominantly female (83.3%), and married/cohabitating (54.1%) and/or had children living at home (54.1%) in about half of cases. On average, they worked 89.2% (*SD* = 14.4%) of fulltime equivalent and had nearly ten years of work experience as nurses (mean = 9.4 years; *SD* = 7.7 years).

### Differences between blue-depleted and standard hospital light environments

Table 1 shows the results from the fixed-effects linear models comparing the effect of BDLE with that of STLE on the outcome variables. Analyses of the work diaries showed within-subject differences of increased subjective sleepiness (by 17%) during evening shifts in the BDLE compared with the STLE (*p* = 0.034; Cohen’s *d* = 0.49) and a 0.2 increase in number of caffeinated beverages consumed during nights in the STLE compared with the BDLE (*p* = 0.027; Cohen’s *d* = 0.37). There were no differences in terms of stress levels, or positive and negative feelings during shifts (p-values ranging from 0.246 to 0.943). Sleep diary data indicated no differences on any of the outcome variables (TIB, TST, SOL, WASO, EMA, SE, number of awakenings, or sleep quality; p-values ranging from 0.206 to 0.991). On actigraphy data, we found no differences on any of the outcome variables (TST, WASO, SPT or SIBS; p-values ranging from 0.129 to 0.949) between conditions.

### Exploratory analyses of questionnaire data

Given the limited sample size available, exploratory one-way between-group ANOVAs were performed on items extracted from the self-rated questionnaires (i.e., outcomes without multiple observations). Supplementary Table 1 shows the means and standard deviations for these variables for all participants and then categorized according to light environment. There were no significant differences between the groups except that nurses working in the BDLE reported perceiving the lighting as warmer (*p* = 0.009) and more relaxing (*p* = 0.023) than nurses in the STLE.

## Discussion

The main aim of the present study was to investigate the effects of a BDLE compared with a STLE in an acute psychiatric unit at St. Olavs Hospital on shift working nurses’ sleep, mood, levels of stress, and caffeine use. To the best of our knowledge, this is the first study to examine how nurses experience working in a BDLE compared with a STLE. Overall, the results showed that most aspects of the nurses’ sleep and functioning were unchanged by exposure to the two light environments. However, the nurses reported higher levels of sleepiness during evening shifts in the BDLE than in the STLE. In addition, nurses reported consuming a slightly higher number of caffeinated beverages during night shifts in the STLE than in the BDLE.

The fact that subjective sleepiness was higher during evening shifts in the BDLE makes sense considering that lower levels of white light exposure during the evening (when it is becoming gradually darker outside) are associated with lower levels of melatonin suppression [47]. Melatonin is a hormone that helps the body to know when it is time to sleep and wake up and melatonin suppression is associated with shifting of the circadian phase so that sleepiness and sleep occur later in the day [48, 49]. An at least a partial circadian adaptation to night shifts might be desirable, since it could potentially increase job performance and reduce the risk of accidents at work or during commute as well as improve daytime sleep during days off work, when nurses must re-adapt to a daytime schedule [50–52]. We did not find support for differences in adaptation to shift work between the light environments, but the lower levels of evening sleepiness in the STLE are likely a reflection of white light having a direct activating effect increasing alertness [3, 40, 41]. As such, increased sleepiness in the BDLE may compromise the nurses’ safety if they, for example, need to react instantly to adverse events. Further, it is comparably surprising that we did not find the same increase in sleepiness during night shifts in the BDLE. One explanation may be that the study does not have statistical power to detect small within-subject effects of BDLE compared with STLE. Another possible explanation is the ceiling effect, whereby the level of sleepiness during the night shift was high in both light environments and that the measurement of sleepiness used in this study is not sufficiently suited to distinguish between the nuances in the levels of sleepiness.

Our research group previously conducted a pilot study with 12 healthy adults using a randomized cross-over design comparing the effects of a BDLE with a STLE on sleep, subjective sleepiness, and the experience of side effects [31]. In that study, the effects of the BDLE were generally positive. Participants did not report higher levels of sleepiness or negative side effects, the participants’ sleep–wake cycle was phase-advanced (i.e., higher levels of melatonin earlier at night), and they slept marginally longer (8.1 min) after residing in the BDLE. One explanation for the conflict with our finding of increased sleepiness during evening shifts in the BDLE could be the difference between assessments, i.e., hourly ratings of sleepiness from 19:00 to 23:00 versus retrospective global assessment of sleepiness during whole shifts. Alternatively, differences in the demands made on participants simply residing in the building compared with nurses performing work-related tasks could further impact the extent to which an individual experiences fatigue and sleepiness. Additionally, in the present study, we did not find significant differences on any sleep outcomes after working in the BDLE compared with the STLE, which might also be explained by the difference between assessments (i.e., polysomnography data versus diary and actigraphy data) or that artificial light exposure when commuting home from work and at home before bedtime is sufficient to reverse the subtle effects of a BDLE at work [53–55].

We also found a small increase in the number of caffeinated beverages consumed (0.2) when undertaking night shifts in the STLE compared with the BDLE. Unfortunately, our data does not allow us to conclude anything about the reason for these findings. For example, it is unclear whether this finding is best explained by differences in participants’ energy levels, their perceived alertness during night shifts across the different light environments, or individual fluctuation in caffeine intake (related to spurious factors), among others. In a review study on the effects of caffeine used as a risk prevention measure among shift workers, it was found that caffeine improved cognitive functioning and reduced the number of performance errors due to sleepiness [56]. Although there were no significant differences in caffeine consumption across the light environments during evening shifts in the present study, it is conceivable that if nurses working evening shifts in the BDLE would increase their consumption of caffeine, this would improve their performance and reduce sleepiness [57].

Interestingly, we found no differences between BDLE and STLE on nurses’ reported levels of stress, positive or negative mood during shifts, or sleep patterns after each shift was completed. This was unexpected given that shift work and, particularly, exposure to blue light frequencies during the dark period of the day, is a known risk factor for poor sleep [6], medical or mental health problems [4], and impaired attention and alertness during waking hours [58]. Some of the negative effects of shift work can generally be attributed to suboptimal shifting of the circadian phase [59], either the absence of a circadian shift when engaging in shift work or a too abrupt circadian shift which potentially could lead to increased irregularity and/or fragmentation of an individual’s sleep and wakefulness pattern (which in turn could affect medical and mental health). However, as light exposure by itself could facilitate shifting of the circadian phase to better adapt to night work [60], we might expect BDLE, or ‘virtual darkness’ [61, 62], to have a different effect on nurses’ sleep and functioning in a naturalistic setting than STLE. In many respects, the limited number of macro-level differences between the light environments is encouraging, as it indicates that a BDLE is not associated with major side effects or harmful effects. However, these findings need to be confirmed in further studies before BDLEs can be clearly established as beneficial to patients and not harmful to clinical staff working in inpatient units.

### Limitations and future directions

There are some important limitations of the present study that should be considered. Due to the comparatively small sample size, we were limited in the types of analyses we were able to conduct. Further, we did not perform any correction for multiple comparisons (e.g., by adjusting for false discovery rate [63]) as this, given our limited sample size, would have increased the risk of false negatives. Failure to detect side effects of the light environments would be potentially harmful to the nurses. A larger sample size is important for reliable, and meaningful multivariable analysis of the effects of a BDLE compared with a STLE on nurses’ sleep, health, and functioning or effects of switching between light environments (including the impact of covariates). A larger sample size would also facilitate analysis of whether the BDLE directly affects the nurses’ circadian rhythms and the impact of possible confounding factors (e.g., if patients exposed to the BDLE were calmer and, as such, influenced the nurses), as well as the potential impact on patient safety associated with a slight increase in sleepiness during evening shifts in the BDLE. Further, although the majority of the assessments included in the present study are validated in the general population, they have not been validated in representative samples of nurses. The within-subject design in a naturalistic setting is a strength of the present study, but due to the low participation rate (29.1%), we cannot ascertain whether our findings are representative of all nurses working at the acute psychiatric unit at St. Olavs Hospital. Although other single-site studies in small workplaces (less than ~ 100 employees) will also necessarily be bound by an upper limit of available participants, having high participation rates would ensure that any drawn conclusions will be representative of all employees. Additionally, use of employer or registry data in future studies, as opposed to self-reporting, could both ease time demands on the participants in addition to serving as a source of objective, high-quality information on how shift working nurses are affected by a BDLE. Such sources could be used to collect information on for example sickness absence, other types of leave, healthcare resource use, and medical or mental health diagnoses. They will not, however, be suitable to investigate individual experiences of day-to-day life, and important information on personal experiences in and of the work environment, levels of presenteeism (i.e., reduced productivity while at work), impairment in general activities outside of work, or subclinical symptoms of medical or mental health conditions.

## Conclusions

Our study suggests that working in a BDLE does not considerably impact the nurses’ sleep, levels of stress, or mood in a naturalistic setting. There was some indication that the light environments may affect the nurses’ functioning during shifts. Limitations of the present study placed restrictions on the analyses that were able to be conducted and the conclusions that could be drawn. We are, however, optimistic that BDLEs in hospitals are acceptable to the nurses. We recommend further investigations on this topic before BDLEs are implemented as standard solutions in healthcare institutions, and we have proposed specific suggestions for designing future large-scale trials and cohort studies.

**Table 1:** Results from fixed effects linear models comparing the effects of BDLE with STLE

	**Mean** **STLE**	**Mean** **BDLE**	**Estimated mean** **Difference**	**95% CI**	**P Value**	**Cohen’s *d***
**Work Diary**						
Number of Caffeinated drinks						
E vs E	2.15	1.19	0.21	-0.42 to 0.84	0.508	0.636
N vs N	2.11	1.73	-0.37	-0.70 to -0.04	0.027*	0.367
E.N. vs E.N	2.14	1.49	-0.09	-0.42 to 0.24	0.582	0.490
How stressful was your shift? *(range 1–5) *^*a*^						
E vs E	2.56	2.31	-0.32	-0.92 to 0.27	0.277	0.225
N vs N	2.95	2.70	-0.30	-0.88 to 0.28	0.312	0.248
E.N. vs E.N	2.74	2.53	-0.24	-0.65 to 0.17	0.246	0.202
How sleepy were you during the shift? *(range 1–5) *^*a*^						
E vs E	2.46	3.03	0.74	0.06 to 1.42	0.034*	0.486
N vs N	2.86	3.07	0.22	-0.34 to 0.77	0.440	0.204
E.N. vs E.N	2.65	3.06	0.42	0.002 to 0.05	0.048	0.372
How positive did you feel during the shift? *(range 1–5) *^*a*^						
E vs E	3.81	3.84	0.10	-0.33 to 0.53	0.654	-0.037
N vs N	3.25	3.53	-0.13	-0.49 to 0.23	0.486	-0.382
E.N. vs E.N	3.55	3.67	-0.34	-0.30 to 0.24	0.804	-0.150
How negative did you feel during the shift? *(range 1–5) *^*a*^						
E vs E	1.62	1.63	0.10	-0.45 to 0.65	0.723	-0.011
N vs N	1.70	1.57	-0.09	-0.46 to 0.28	0.619	0.194
E.N. vs E.N	1.66	1.60	-0.01	-0.32 to 0.30	0.943	0.076
**Sleep Diary**						
Time in Bed, min						
E vs E	507.00	481.00	31.47	-198.37 to 261.30	0.785	0.088
N vs N	488.00	529.00	98.99	-177.39 to 375.37	0.476	-0.104
E.N. vs E.N	499.00	508.00	73.34	-102.50 to 249.17	0.410	-0.025
Sleep Period Time, min						
E vs E	410.00	405.00	-18.19	-70.74 to 34.37	0.491	0.074
N vs N	361.00	399.00	65.30	-45.14 to 175.73	0.241	-0.238
E.N. vs E.N	390.00	401.00	34.18	-28.13 to 96.48	0.279	-0.093
Total Sleep Time, min						
E vs E	356.00	371.00	-7.57	-61.88 to 46.74	0.781	-0.203
N vs N	328.00	376.00	6.11	-41.30 to 173.52	0.223	-0.314
E.N. vs E.N	344.00	374.00	39.25	-21.84 to 100.33	0.206	-0.239
Sleep Onset Latency, min						
E vs E	23.31	24.54	-3.09	-20.29 to 14.11	0.720	-0.046
N vs N	14.68	8.85	-2.25	-13.15 to 8.65	0.680	0.334
E.N. vs E.N	19.64	15.94	-2.82	-12.32 to 6.67	0.557	0.162
Wake After Sleep Onset, min						
E vs E	34.07	9.11	-10.80	-34.95 to 13.36	0.374	0.804
N vs N	18.65	13.62	1.43	-14.32 to 17.19	0.856	0.194
E.N. vs E.N	27.43	11.58	-3.58	-17.08 to 9.92	0.600	0.537
Early Morning Awakening, min						
E vs E	15.74	11.04	-5.02	-16.61 to 6.57	0.389	0.287
N vs N	67.68	114.00	54.63	-129.70 to 238.96	0.555	-0.172
E.N. vs E.N	37.50	67.68	28.12	67.21 to 123.45	0.560	-0.156
Sleep Efficiency, %						
E vs E	77.84	83.83	0.66	11.48 to 12.80	0.913	-0.378
N vs N	78.31	79.79	0.09	-11.44 to 11.63	0.987	-0.083
E.N. vs E.N	78.03	81.61	0.67	-7.39 to 8.73	0.870	-0.214
Nightly Awakenings, No						
E vs E	1.60	1.39	-0.41	-1.26 to 0.44	0.336	0.152
N vs N	1.26	1.35	0.38	-0.37 to 1.12	0.313	-0.068
E.N. vs E.N	1.45	1.37	0.05	-0.50 to 0.59	0.864	0.060
Sleep Quality ^*a*^						
E vs E	3.05	3.48	0.23	-0.65 to 1.11	0.601	-0.397
N vs N	3.29	3.29	< 0.00	-0.52 to 0.52	0.991	0.000
E.N. vs E.N	3.15	3.38	0.10	-0.37 to 0.57	0.671	-0.243
**Actigraphy**						
Sleep Period Time, min						
E vs E	410.00	402.00	11.65	-48.65 to 71.95	0.698	0.123
N vs N	369.00	389.00	67.18	-53.89 to 188.26	0.269	-0.135
E.N. vs E.N	393.00	395.00	53.19	-15.89 to 122.28	0.129	-0.020
Total Sleep Time, min						
E vs E	362.00	345.00	1.86	-56.37 to 60.09	0.949	0.283
N vs N	342.00	366.00	68.88	-42.37 to 180.13	0.218	-0.329
E.N. vs E.N	353.00	356.00	50.30	-14.77 to 115.37	0.128	-0.221
Wake After Sleep Onset, min						
E vs E	52.00	57.24	7.72	-16.28 to 31.70	0.518	-0.131
N vs N	26.55	22.89	-1.79	-22.26 to 18.67	0.860	0.126
E.N. vs E.N	40.57	39.40	2.15	-12.57 to 16.86	0.772	0.031
Sleep Periods, No						
E vs E	14.10	13.79	-2.23	-5.13 to 0.68	0.130	0.070
N vs N	11.67	11.76	1.64	-2.86 to 6.14	0.464	-0.017
E.N. vs E.N	13.10	12.84	0.13	-2.45 to 2.71	0.919	0.053

*BDLE* Blue-depleted light environment, *STLE*   Standard hospital light environment, *E*  Evening shifts, *N*  Night shifts; *E*.*N*. evening/night shifts combined. Number of observations = 168; *n* = 25

^a^ Rated on a 5-point scale from not at all stressed/sleepy/positive/negative/restless (1) to very stressed/sleepy/positive/negative/restless (5)

## Supplementary Information

**Additional file 1: Supplementary Table 1**. Results from one-way between groups ANOVAs of questionnaire data.

## Data Availability

The datasets generated and analyzed during the current study are not publicly available as we do not have ethical approval for this type of data sharing.

## Abbreviations

BAL: Evaluation about the beliefs about the light condition
BDLE: Blue-depleted light environment
EMA: Early Morning Awakening
H&ES: Headache and Eyestrain Scale
K10: The Kessler Psychological Distress Scale
PHQ: Psychological Health Questionnaire
REK: Regional Ethical Committee of Central Norway
SE: Sleep Efficiency
SIBS: Number of sleep periods
SOL: Sleep Onset Latency
SPT: Sleep Period Time
STLE: Standard hospital light environment
TIB: Time in Bed
TST: Total Sleep Time
WASO: Wake After Sleep Onset
