# Supplementary material for: Sleep and work functioning in nurses undertaking inpatient shifts in a blue-depleted light environment
Source: BMC Nurs. 2022 Jul 18;21:187. doi: 10.1186/s12912-022-00973-4 (PMC9290304; doi:10.1186/s12912-022-00973-4)
Supplement: Supplementary file 1 — Additional file 1: Supplementary Table 1. Results from one-way between groups ANOVAs of questionnaire data. [file 12912_2022_973_MOESM1_ESM.docx]

| **Supplementary Table 1**. Results from one-way between groups ANOVAs of questionnaire data. | | | | |
| --- | --- | --- | --- | --- |
|  | **Blue-depleted (n = 9)** | **Standard (n = 15)** | **P value** | **Overall** |
| **Kessler Psychological Distress Scale**, total | 18.00 (3.46) | 16.20 (2.75) | .173 | 16.88 (3.10) |
| Tired *(range 1-5) ^a^* | 2.33 (.87) | 2.07 (.88) | .479 | 2.17 (.87) |
| Nervous | 1.56 (1.13) | 1.60 (.51) | .895 | 1.58 (.78) |
| Nervous & unable to calm down | 1.00 (.00) | 1.00 (.00) | . | 1.00 (.00) |
| Hopeless | 1.33 (.50) | 1.33 (.49) | 1.00 | 1.34 (.48) |
| Restless | 1.78 (.83) | 1.60 (.74) | .591 | 1.67 (.76) |
| Restless & unable to sit still | 1.44 (.73) | 1.20 (.41) | .302 | 1.29 (.55) |
| Depressed | 1.67 (.71) | 1.27 (.46) | .105 | 1.42 (.58) |
| Fatigued | 1.89 (.93) | 1.67 (.62) | .487 | 1.75 (.74) |
| Sadness | 1.11 (.33) | 1.00 (.00) | .203 | 1.04 (.20) |
| Worthless | 1.56 (.73) | 1.13 (.35) | .067 | 1.29 (.55) |
| **Psychological Health Questionnaire** |  |  |  |  |
| Trouble sleeping *(range 1-7) ^b^* | 3.33 (.2.18) | 2.93 (1.22) | .568 | 3.08 (1.61) |
| Awake at night | 4.33 (1.87) | 4.07 (1.49) | .703 | 4.17 (1.61) |
| Nightmares or bad dreams | 1.89 (.93) | 1.87 (1.55) | .969 | 1.87 (1.33) |
| Restful sleep*^bb^* | 4.00 (.50) | 3.80 (.49) | .777 | 3.87 (1.62) |
| Headache | 2.44 (1.42) | 2.00 (.93) | 362 | 2.17 (1.13) |
| Headache due to stress | 2.33 (1.41) | 2.33 (1.54) | 1.00 | 2.33 (1.47) |
| Headache due to frustration | 1.89 (1.45) | 1.53 (.74) | .434 | 1.67 (1.05) |
| Upset stomach | 2.22 (1.99) | 1.93 (1.34) | .673 | 2.04 (1.57) |
| Careful with food to avoid upset stomach | 2.44 (2.40) | 1.87 (1.60) | .485 | 2.08 (1.91) |
| Nausea | 1.67 (1.66) | 1.40 (1.30) | .665 | 1.50 (1.41) |
| Constipation/diarrhea | 2.00 (2.12) | 2.00 (1.41) | 1.00 | 2.00 (1.67) |
| Mild cold, times | 2.33 (2.18) | 1.27 (.46) | .077 | 1.67 (1.44) |
| Severe cold, times | 1.00 (.00) | 1.07 (.26) | .451 | 1.04 (.20) |
| Duration of cold | 3.67 (3.01) | 2.00 (1.27) | .125 | 2.59 (2.12) |
| **Headache and Eyestrain Scale**, total | 15.56 (4.78) | 14.40 (3.56) | .505 | 14.83 (3.996) |
| Irritability *(range 1-4) ^c^* | 2.11 (.79) | 1.87 (.83) | .484 | 1.96 (.81) |
| Headache | 1.89 (.93) | 1.73 (.88) | .686 | 1.79 (.88) |
| Eye strain | 2.22 (.97) | 2.13 (.99) | .832 | 2.17 (.96) |
| Eye discomfort | 2.11 (1.05) | 1.73 (.80) | .330 | 1.87 (.90) |
| Eye fatigue | 2.22 (.97) | 2.00 (.85) | .561 | 2.08 (.88) |
| Difficulty focusing | 1.89 (.93) | 1.80 (.68) | .789 | 1.83 (.76) |
| Difficulty concentrating | 1.89 (.78) | 1.80 (.68) | .771 | 1.83 (.70) |
| Blurred vision | 1.22 (.44) | 1.33 (.49) | .582 | 1.29 (.46) |
| **Evaluation about beliefs about the light condition** |  |  |  |  |
| Unpleasant - pleasant *(range 1-7) ^d^* | 4.56 (1.42) | 4.33 (1.54) | .729 | 4.42 (1.47) |
| Uncomfortable - comfortable | 4.00 (2.37) | 4.40 (1.55) | .609 | 4.25 (1.80) |
| Disturbing - not disturbing | 4.33 (2.50) | 5.07 (1.87) | .421 | 4.79 (2.11) |
| Causing glare - not causing glare | 4.89 (2.09) | 4.80 (1.90) | .916 | 4.83 (1.92) |
| Uniform - non-uniform | 4.56 (1.94) | 5.67 (1.45) | .123 | 5.25 (1.70) |
| Warm - cold | 2.89 (1.76) | 4.87 (1.55) | **.009*** | 4.12 (1.87) |
| Dim - bright | 3.33 (2.35) | 4.67 (1.84) | .135 | 4.17 (2.099) |
| Relaxing - stimulating | 2.56 (2.01) | 4.47 (1.77) | **.023*** | 3.75 (2.05) |
| Unsuitable for work - suitable for work *(range 1-7) ^d^* | 3.78 (1.86) | 4.93 (1.44) | .101 | 4.50 (1.67) |
| Work strain *(range 1-5) ^e^* | 2.22 (.97) | 2.57 (.94) | .400 | 2.43 (.945) |
| **Performance and Effort** |  |  |  |  |
| Concentration *(range 1-7) ^f^* | 5.22 (.83) | 5.64 (.50) | .143 | 5.48 (.665) |
| Performance | 5.11 (1.05) | 5.50 (.52) | .249 | 5.35 (.775) |
| Effort | 4.78 (.83) | 4.43 (1.09) | .423 | 3.43 (.99) |
| **Other side effects** |  |  |  |  |
| Dry eyes, mouth, or nose *(range 1-4) ^c^* | 2.11 (1.05) | 1.93 (1.10) | .701 | 2.00 (1.06) |
| Sleepiness during the day | 2.78 (1.09) | 2.33 (.72) | .242 | 2.50 (.88) |
| Poor sleep quality | 2.44 (1.33) | 2.20 (.78) | .573 | 2.29 (.99) |
| Too much sleep | 1.00 (.00) | 1.27 (.46) | .097 | 1.17 (.38) |
| Tired during the day | 3.11 (.93) | 2.33 (.98) | .067 | 2.63 (1.01) |
| Restlessness | 1.67 (.71) | 1.47 (.74) | .523 | 1.54 (.72) |
| Dizziness | 1.44 (1.01) | 1.13 (.35) | .285 | 1.25 (.67) |
| Excessive sweat | 1.22 (.44) | 1.47 (.64) | .325 | 1.38 (.57) |
| Diarrhea | 1.33 (1.00) | 1.13 (.35) | .483 | 1.21 (.65) |
| Changed/poor appetite | 1.11 (.33) | 1.20 (.78) | .749 | 1.17 (.64) |
| Constipation | 1.22 (.44) | 1.40 (.91) | .591 | 1.33 (.76) |
| Nausea/upset stomach | 1.44 (1.01) | 1.20 (.56) | .452 | 1.29 (.75) |
| **Morningness-Eveningness Questionnaire** *^g^* | 14.75 (2.38) | 14.53 (6.10) | .925 | 14.61 (5.05) |
| Means, standard deviations are shown for the blue-depleted and standard hospital light environment separately, and for participants overall from the first round of data collection. ^a^ Rated on a 5-point scale from ‘not at all’ (1) to ‘most of the time’ (5); ^b^ Rated on a 7-point scale from ‘not at all’ (1) to ‘all the time’ (7); ^bb^ Scoring on this item has been reversed; ^c^ Rated on a 4-point scale from ‘absent’ (1) to ‘severe’ (4); ^d^ Rated on a 7-point scale with each word pair as anchoring; ^e^ Rated on a 5-point scale from ‘not at all heavy’ (1) to ‘very heavy’ (5); ^f^ Rated on a 7-point scale so that higher score reflect degree of each item; *^g^* Scores on average reflect neither morning or evening types, with higher degree of chronotype variance in the standard hospital light environment. | | | | |
